# CT-based deep learning model: a novel approach to the preoperative staging in patients with peritoneal metastasis

**DOI:** 10.1007/s10585-023-10235-5

**Published:** 2023-10-05

**Authors:** Jipeng Wang, Yuannan Hu, Hao Xiong, Tiantian Song, Shuyi Wang, Haibo Xu, Bin Xiong

**Affiliations:** 1 Department of Gastrointestinal Surgery, Zhongnan Hospital of Wuhan University, No.169 Donghu Road, Wuhan, 430071 Hubei China; 2 Hubei Key Laboratory of Tumor Biological Behaviors, No.169 Donghu Road, Wuchang District, Wuhan, 430071 China; 3 Department of Radiology, Zhongnan Hospital of Wuhan University, Wuhan, 430071 China; 4 Department of information Center, Zhongnan Hospital of Wuhan University, Wuhan, 430071 China

**Keywords:** Peritoneal metastasis, Deep learning, Peritoneal carcinomatosis index, Radiomics, Enhanced computed tomography

## Abstract

Peritoneal metastasis (PM) is a frequent manifestation of advanced abdominal malignancies. Accurately assessing the extent of PM before surgery is essential for patients to receive optimal treatment. Therefore, we propose to construct a deep learning (DL) model based on enhanced computed tomography (CT) images to stage PM preoperatively in patients. All 168 patients with PM underwent contrast-enhanced abdominal CT before either open surgery or laparoscopic exploration, and peritoneal cancer index (PCI) was used to evaluate patients during the surgical procedure. DL features were extracted from portal venous-phase abdominal CT scans and subjected to feature selection using the Spearman correlation coefficient and LASSO. The performance of models for preoperative staging was assessed in the validation cohort and compared against models based on clinical and radiomics (Rad) signature. The DenseNet121-SVM model demonstrated strong patient discrimination in both the training and validation cohorts, achieving AUC was 0.996 in training and 0.951 validation cohort, which were both higher than those of the Clinic model and Rad model. Decision curve analysis (DCA) showed that patients could potentially benefit more from treatment using the DL-SVM model, and calibration curves demonstrated good agreement with actual outcomes. The DL model based on portal venous-phase abdominal CT accurately predicts the extent of PM in patients before surgery, which can help maximize the benefits of treatment and optimize the patient’s treatment plan.

**Graphical abstract:**

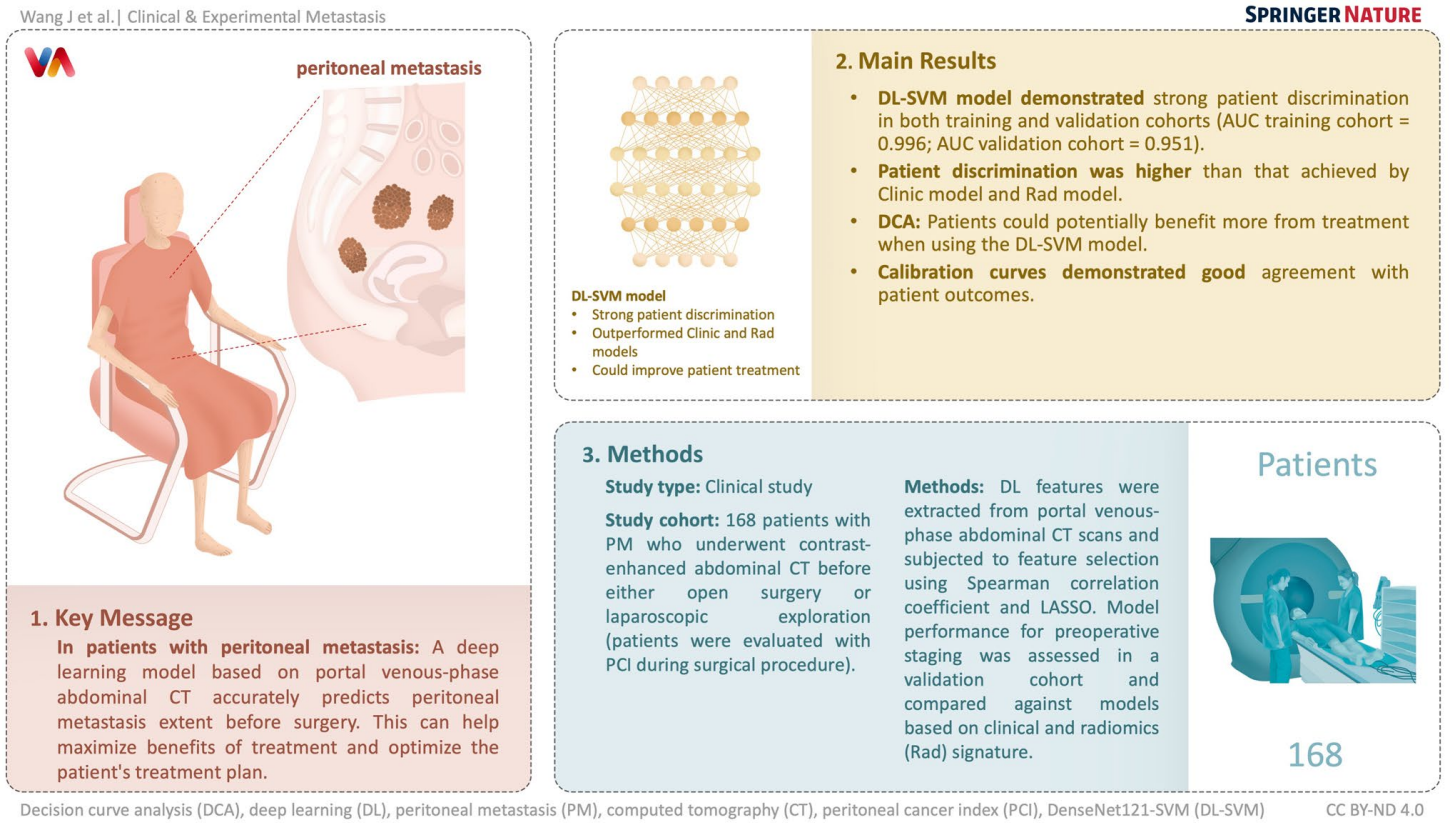

**Supplementary Information:**

The online version contains supplementary material available at 10.1007/s10585-023-10235-5.

## Introduction

Peritoneal metastasis (PM) is the most common metastasis in advanced abdominal tumors, with a median survival of no more than 12 months in some patients, and a worse prognosis than other metastasis, such as the liver or lung [1, 2]. In recent years, cytoreductive surgery (CRS) combined with hyperthermic intraperitoneal chemotherapy (HIPEC) has been widely accepted for treatment of PM, emerging as the most effective approach to prolonging survival in the majority of cases [3, 4]. CRS + HIPEC has been endorsed by the Peritoneal Surface Oncology Group International (PSOGI) as the established standard of care for patients diagnosed with peritoneal pseudomyxoma, malignant peritoneal mesothelioma, and colorectal peritoneal metastases. Furthermore, it is the recommended therapeutic approach for patients with peritoneal metastases associated with gastric and ovarian cancers [5–10].

However, not all patients may experience positive outcomes from CRS + HIPEC treatment. The peritoneal carcinomatosis index (PCI), together with the cytoreductive completeness score (CCS), has been shown to be a major determinant of overall patient survival [11–13]. Patients with severe PM often have a relatively poor prognosis and are unlikely to achieve a clinical cure after CRS treatment (CCS of 0 or 1). For instance, in colorectal cancer patients, PCI > 20 is considered a contraindication to CRS + HIPEC treatment [14], while gastric cancer patients with normal tumor markers and PCI ≤ 16 are more likely to benefit from surgical treatment [15]. Currently, the gold standard for assessing PCI is laparoscopic [16], and some patients with extensive metastasis may have to forgo further surgical treatment after the invasive examination. Open-close surgery has been reported in up to 23.4% of cases [17].

Imaging examinations such as CT and MRI have become common preoperative procedures for patients with PM. While they can provide information about the presence and extent of metastasis, their low sensitivity may result in discrepancies between the imaging results and the actual condition of the patient. As a result, CT-PCI and MRI-PCI demonstrate limited ability to stratify patients for selection when compared to surgical PCI (S-PCI), with a concordance index of only 0.47–0.79 [18–21]. Furthermore, the unique anatomical characteristics of the peritoneum pose a significant challenge in distinguishing it from surrounding visceral fat on imaging, thereby necessitating a higher level of expertise from the radiologist.

Radiomics (Rad) is a promising image data analysis method that has emerged in recent years, capable of quantifying traditional medical images into structured data features and interpreting images from multiple dimensions [22]. It has demonstrated potential in determining the benignity and malignancy of tumors, staging, and prognosis [23–25]. Similarly, deep learning (DL) can also extract quantified features from images with higher dimensionality and more accurate performance than Rad features (RF) [26]. Previous studies have shown that DL exhibits better performance than RF in predicting preoperative occult PM or lymphatic metastasis in gastric cancer patients [27, 28]. However, there are currently no reported studies utilizing DL for the preoperative staging of PM patients.

In summary, the purpose of this study was to develop and validate a DL model for the preoperative staging of PM patients to individualize patient treatment based on accurate staging.

## Materials and methods

### Patient enrolment

**Fig. 1:**
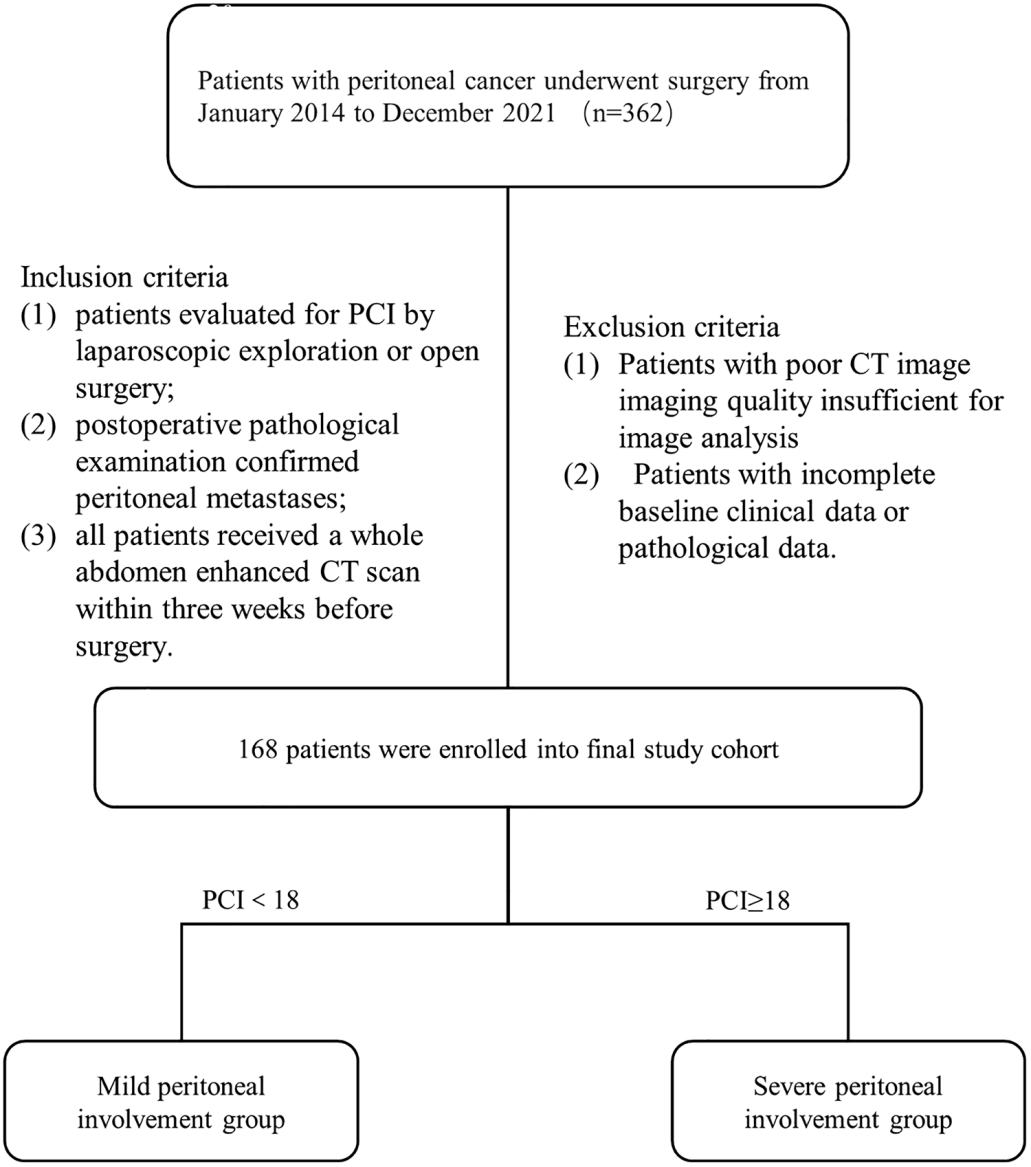
Flow chart of the study cohort recruitment

This study included 362 PM patients who underwent surgery at Zhongnan Hospital of Wuhan University from January 2014 to December 2021. The inclusion criteria for patients were as follows: (1) evaluation of PCI via laparoscopic exploration or open surgery, (2) confirmation of PM through postoperative pathological examination, and (3) receipt of a whole contrast-enhanced abdominal CT within three weeks prior to surgery. Patients were excluded if they had poor CT imaging quality or incomplete baseline clinical or pathological information. Of the initial pool of patients, 168 patients were included in the final analysis and categorized into two groups: Mild peritoneal involvement (MPI) group (PCI < 18) and Heavy peritoneal involvement (HPI) group (PCI ≥ 18), based on the S-PCI. This division considered the established indications for gastrointestinal CRS, which typically fall below the range of 16–20 for PCI. The training and validation cohorts were randomly divided into an 8:2 ratio. A flow chart of this study design is provided in Fig. 1.

The Medical Ethics Committee of Wuhan University Zhongnan Hospital approved this study. Since this study was retrospective, informed consent from patients was waived.

### Intraoperative evaluation

The S-PCI was determined during laparoscopy exploration or open surgery by a team of surgeons. The abdominopelvic cavity was partitioned into 13 distinct regions, and the size of the tumor in each region was used to assign a score, with a score of 0 indicating no tumor, 1 for tumor diameter < 0.5 cm, 2 for tumor diameter ranging from 0.5 to 5 cm, and 3 for tumor diameter exceeding 5 cm. The summation of scores from all regions yielded the S-PCI, which could range from 0 to 39 [29]. All samples obtained during surgery were confirmed as PM through postoperative pathological examination.

### CT image acquisition

CT examinations were conducted on 128-slice (Siemens SOMATOM Definition CT) and 64-channel (GE Discovery CT750, Philips Ingenuity CT) scanners with patients in the supine position following a 6-h fasting period. Patients were trained to control their breathing before the scan to minimize any breathing-related artifacts. The CT scans encompassed the region extending from the diaphragm to the bony pelvic floor. Prior to undergoing the contrast-enhanced CT examination, patients were administered contrast agents (5.300 mL/kg, iohexol 40 mg I/mL) via the anterior elbow vein at a rate of 5.1 mL/s. The following parameters were used for the CT scan: tube current ranging from 150 to 350 mA, tube voltage of 120 kVp, field of view spanning 30–45 cm, matrix size of 512 × 512, and reconstructed slice thickness between 1 and 5 mm.

### Image preprocessing and segmentation

**Fig. 2:**
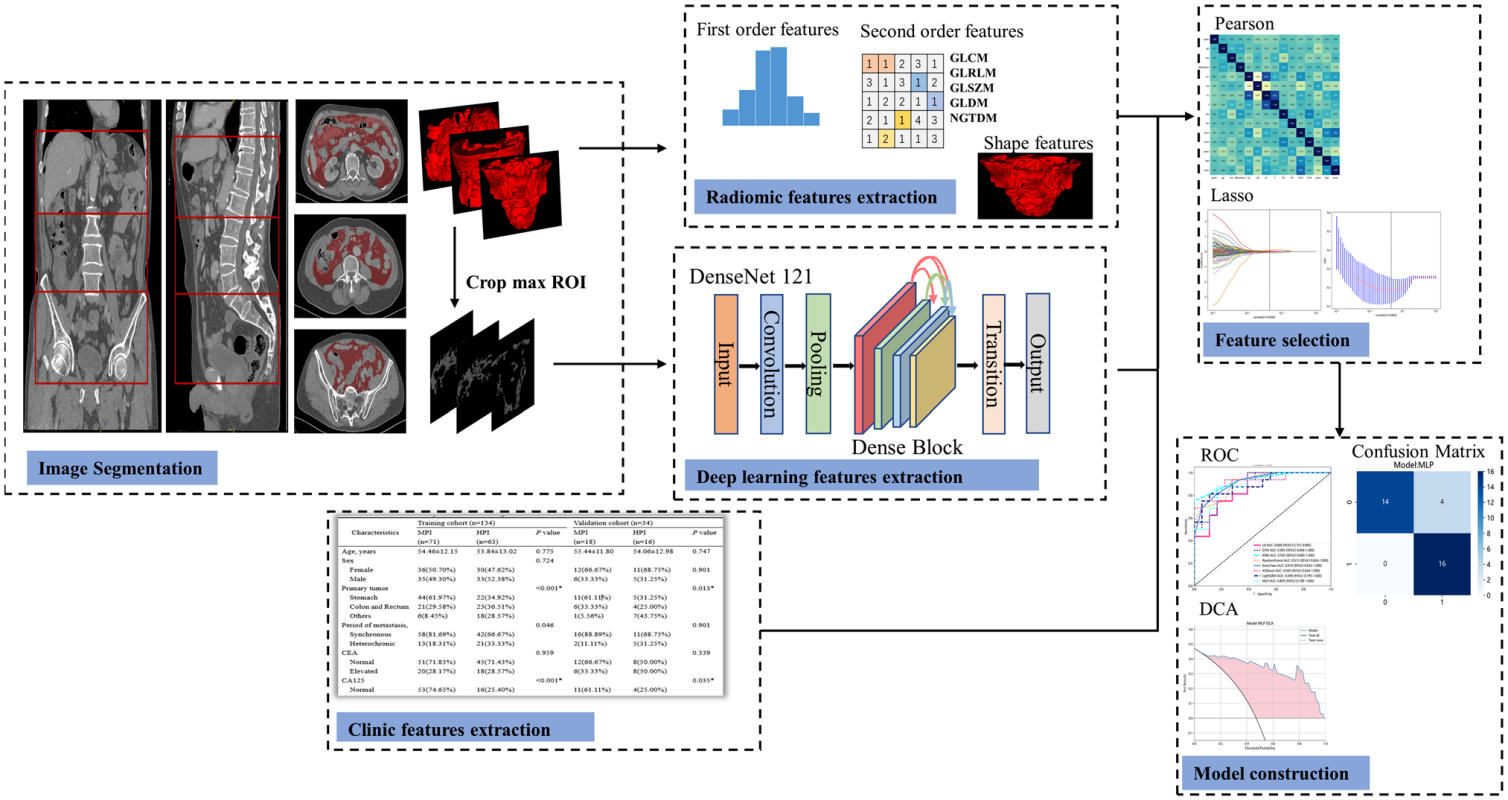
The workflow in this study. Firstly, the clinical and imaging data of the patients were collected. Subsequently, ITK-SNAP was employed to outline the imaging data and obtain the region of interest (ROI). The features of the ROI were then extracted using both radiomics and deep learning techniques. Finally, a machine learning-based peritoneal carcinomatosis index (PCI) prediction model was constructed based on filtered features. The direct predictive ability of different models was compared through evaluation metrics including area under the curve (AUC), confusion matrix, and decision curve analysis (DCA)

We chose CT images of the portal venous-phase for the relevant study because they could well separate between normal tissues, blood vessels, tumors and non-neoplastic organs. To reduce the effect of different volume pixels, we used the nearest interpolation method to resample the voxels to 1 mm × 1 mm × 1 mm. The 3D peritoneal volume of interest (VOI) segmentation was using ITK-SNAP software (v.4.0.0, http://www.itksnap.org). A novel approach to segmenting the 3D peritoneal volume of interest was developed in this study. This was achieved by dividing the entire abdomen into three sections: upper, middle, and lower abdomen. The abdomen was first segmented without organs and organs, musculoskeletal and vascular areas using semi-automatic segmentation on ITK-SNAP (see Supplementary 1 for details), and then by two experienced imaging physicians manually corrected the VOI in the abdominal window (window level: 30HU, window width: 700HU) to obtain the peritoneal VOI of the upper, middle and lower parts. Thirty patients were randomly selected after 30 days to have the VOI segmented again to exclude intra- and interobserver variation. The flow in the whole study was as in Fig. 2.

### Radiomics feature extraction

RF extraction of peritoneal VOI was performed using PyRadiomics (v3.1.0) based on the Python (3.9.7) open-source platform. The types and distribution of RF areshown in the supplementary material. The extraction process aligns with the Imaging Biomarker Standardization Initiative (IBSI) [30]. The reproducibility of RF was assessed by the intraclass correlation coefficient (ICC), and features with ICC ≥ 0.75 proved to have low variance and high reproducibility.

### Deep learning feature extraction

The layer with the largest peritoneal area from the three abdominal VOI of patients were selected as the patient’s DL region of interest (ROI). DenseNet, InceptionV3, ResNet18, ResNet50, and ResNet101 were pre-trained convolutional neural network models based on ImageNet. In the present study, DL models were utilized to extract DL features (DLF) from the ROI. To obtain the DLF, the last fully connected layer of the model was removed and global maximum pooling was applied to extract the maximum value of each feature layer. The original value of the output image features was used as the DLF. To visualize the DLF, the guided class activation map (Grad-CAM) was used. Grad-CAM is a visualization technique utilized to identify the key regions within an input image that significantly influenced the output of the neural network [31]. The PyTorch 1.4.1 library in Python 3.7.0 (https://pytorch.org) was used to implement the neural network.

### Feature selection and model construction

Normalization of data is a common step in machine learning to scale and center the data, which helps in improving the accuracy of the model. In this study, the Z-Score method was used for normalization, which transforms the features to have a mean of zero and standard deviation of one. After normalization, Spearman correlation coefficient was calculated to identify highly correlated features, and one of the features with a strong correlation was retained (correlation coefficient > 0.9). To further select the most representative features, the least absolute shrinkage and selection operator (LASSO) regularization was introduced, which shrinks the regression coefficients towards zero and selects the features with non-zero coefficients. The regularization parameter λ was determined using five-fold cross-validation.

Nine machine learning methods (Table 2) were used to construct the classification model. The training and validation cohorts were randomly split in an 8:2 ratio, and the optimal model was selected based on the area under the receiver operating characteristic (ROC) curve (AUC). Finally, the performance of the model was evaluated using the decision curve analysis (DCA) and calibration curve.

### Statistical analysis

Statistical analyses were performed using the Python 3.9.7 platform (https://www.python.org/). Continuous variables were presented as mean values and standard deviations, and analyzed using either Mann-Whitney U test or Student t-test. Categorical variables were reported as absolute number and analyzed using chi-square test or Fisher’s exact test. The Delong test was utilized to determine the statistical difference between the AUC values. A significance level of 0.05 was chosen for all tests, meaning that a *p* value below this level was considered statistically significant.

## Results

### Clinical characteristics of the patients

In this study, a total of 168 patients were recruited and allocated randomly into either the training cohort (n = 134) or the validation cohort (n = 34) at an 8:2 ratio. Table 1 presents the characteristics of all patients. Among the patients, 79 (47.02%) were male and 89 (52.98%) were female, while 89 (52.98%) belonged to the MPI group, and 79 (47.02%) were in the HPI group (Table 1). The distribution of each clinical information was balanced between the two cohorts. Chi-square and t tests indicated that Primary tumor, CA125, CA199, and L/M were significantly correlated with PCI in patients (*P* < 0.05). Subsequently, univariate and multivariate regression analyses were conducted, revealing that Primary tumor (*P* = 0.004), CA125 (*P* < 0.001), and L/M (*P* = 0.001) could serve as effective diagnostic indicators of PCI staging in patients preoperatively.

**Table 1:** Characteristics of patients

Characteristics	Training cohort (n = 134)			Validation cohort (n = 34)		
	MPI (n = 71)	HPI (n = 63)	*P* value	MPI (n = 18)	HPI (n = 16)	*P* value
Age, years	54.46 ± 12.15	53.84 ± 13.02	0.775	55.44 ± 11.80	54.06 ± 12.98	0.747
Gender			0.724			
Female	36(50.70%)	30 (47.62%)		12 (66.67%)	11(68.75%)	0.901
Male	35(49.30%)	33 (52.38%)		6(33.33%)	5(31.25%)	
Primary tumor			< 0.001*			0.013*
Stomach	44 (61.97%)	22 (34.92%)		11 (61.11%)	5(31.25%)	
Colon and Rectum	21 (29.58%)	23 (36.51%)		6(33.33%)	4(25.00%)	
Others	6(8.45%)	18 (28.57%)		1(5.56%)	7(43.75%)	
Period of metastasis			0.046			0.901
Synchronous	58 (81.69%)	42 (66.67%)		16 (88.89%)	11(68.75%)	
Heterochronic	13 (18.31%)	21 (33.33%)		2(11.11%)	5(31.25%)	
CEA			0.959			0.339
Normal	51 (71.83%)	45 (71.43%)		12 (66.67%)	8(50.00%)	
Elevated	20(28.17%)	18 (28.57%)		6(33.33%)	8(50.00%)	
CA125			< 0.001*			0.035*
Normal	53 (74.65%)	16(25.40%)		11 (61.11%)	4(25.00%)	
Elevated	18(25.35%)	47(74.60%)		7 (38.89%)	12(75.00%)	
CA199			0.020*			0.692
Normal	49 (69.01%)	31 (49.21%)		8 (44.44%)	6(37.50%)	
Elevated	22 (30.99%)	32 (50.79%)		10 (55.56%)	10(62.50%)	
N/L	4.85 ± 12.64	5.31 ± 10.21	0.819	1.83 ± 1.17	2.89 ± 2.20	0.083
L/M	3.21 ± 1.33	2.34 ± 0.98	< 0.001*	3.41 ± 1.24	2.66 ± 1.33	0.099
Pathology			0.277			0.246
Adenocarcinoma	57 (80.28%)	55(87.30%)		17 (94.44%)	13 (81.25%)	
Signet ring and mucinous cell carcinoma	14 (19.72%)	8(12.70%)		1(05.56%)	3(18.75%)	
Differentiation			0.879			0.403
Poor	51 (71.83%)	46 (73.02%)		11 (61.11%)	12(75.00%)	
Moderate and well	20(28.17%)	17 (26.98%)		7 (38.89%)	4(25.00%)	
Ascites			0.767			0.162
No (≤ 300mL)	56 (78.87%)	51(80.95%)		12 (66.67%)	14 (87.50%)	
Yes (> 300mL)	15(21.13%)	12 (19.05%)		6(33.33%)	2(12.50%)	
Surgical PCI	8.80 ± 4.74	26.59 ± 6.89		7.94 ± 3.90	28.38 ± 6.51	

*CEA* carcinoembryonic antigen, *CA* carbohydrate antigen, *N* neutrocyte, *L* lymphocyte, *M* monocyte, *PCI* peritoneal cancer index

*P value < 0.05

### Feature extraction and selection for model construction

**Fig. 3:**
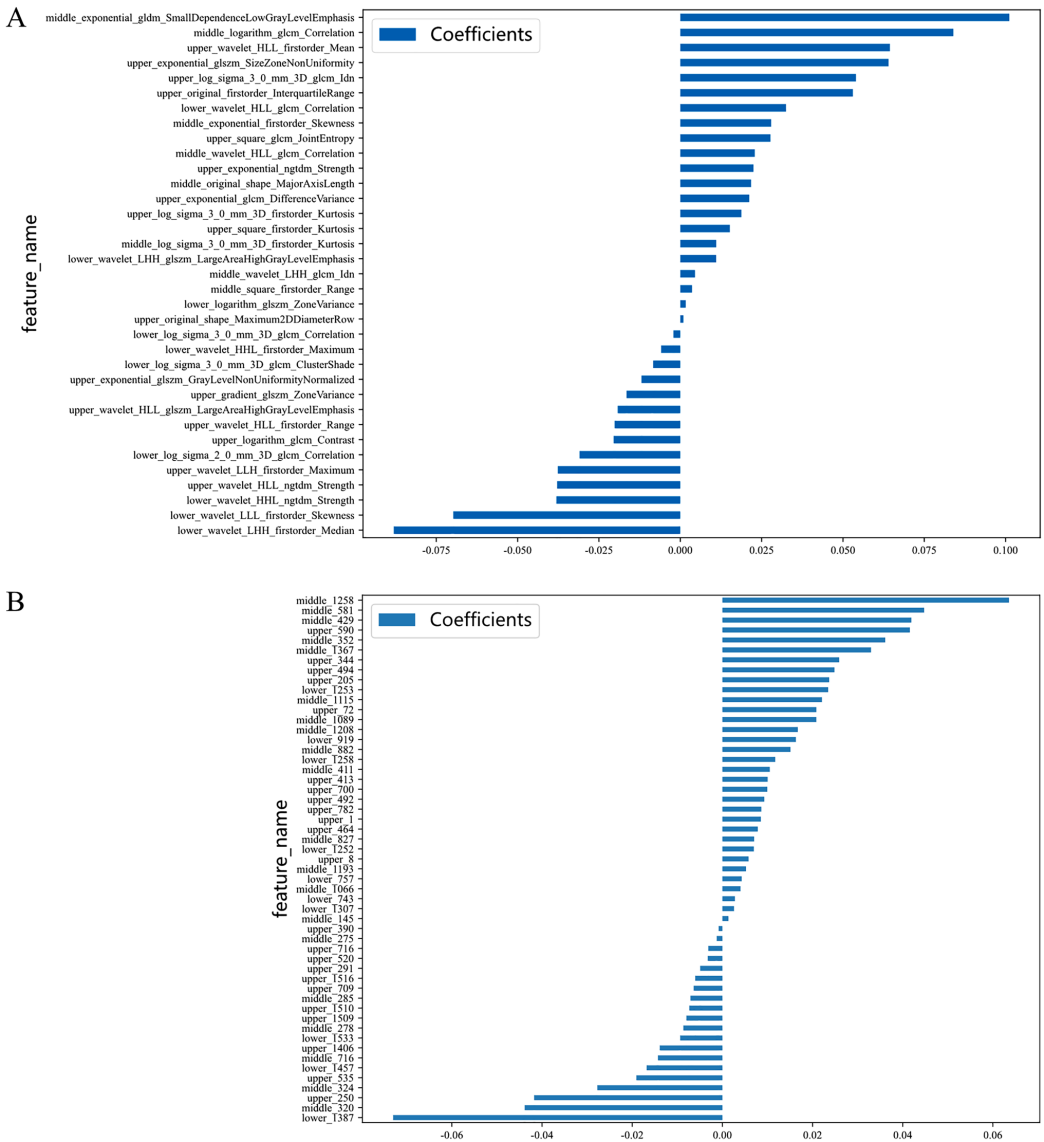
Histogram of filtered feature weighting coefficients. **A** RF; **B** DLF

A total of 4683 RF were extracted from the upper, middle, and lower peritoneal ROIs, with 1561 features in each part. After excluding 1231 features with poor reproducibility (ICC < 0.75) based on ICC reproducibility assessment, 35 RF (as shown in Fig. 3A) were obtained for model construction through further screening with Spearman correlation coefficient and Lasso to exclude features with weak correlation. The Rad-SVM model demonstrated the best prediction performance (as demonstrated in Table 2), and the AUC in the validation cohort was 0.906 (95% CI 0.804–1.000).

Regarding the combination of six DL models and nine machine learning methods used for feature extraction, DenseNet121-SVM demonstrated the best performance (as shown in Table 3), with 4704 DLF extracted (1568 features each in the upper, middle, and lower abdomen). After filtering, 53 DLF were finally selected (as shown in Fig. 3B), and the AUC of the constructed model in the validation cohort was 0.951 (95% CI 0.887–1.000).

**Table 2:** Performance of radiomics models constructed by different machine learning methods

	AUC (95% CI)		Accuracy		Sensitivity		Specificity	
	Training cohort	Validation cohort	Training cohort	Validation cohort	Training cohort	Validation cohort	Training cohort	Validation cohort
LR	0.991 (0.982–1.000)	0.833 (0.684–0.986)	0.955	0.824	0.921	0.812	0.983	0.882
SVM	0.986 (0.974–0.999)	0.906 (0.804–1.000)	0.940	0.882	0.905	0.812	0.972	0.944
KNN	0.922 (0.879–0.964)	0.804 (0.651–0.957)	0.843	0.735	0.762	0.875	0.915	0.611
RandomForest	1.000 (1.000–1.000)	0.842 (0.697–0.988)	1.000	0.824	1.000	0.778	1.000	0.778
ExtraTrees	1.000 (1.000–1.000)	0.830 (0.697–0.963)	1.000	0.735	1.000	0.938	1.000	0.556
XGBoost	1.000 (1.000–1.000)	0.875 (0.758–0.992)	1.000	0.794	1.000	0.875	1.000	0.722
LightGBM	0.973 (0.952–0.994)	0.884 (0.771–0.996)	0.910	0.853	0.937	0.750	0.887	0.944
GradientBoosting	0.997 (0.993–1.000)	0.854 (0.723–0.985)	0.970	0.853	0.968	0.812	0.972	0.889
AdaBoost	0.984 (0.969–0.998)	0.856 (0.719–0.993)	0.940	0.824	0.889	0.750	0.986	0.889

*AUC* area under the receiver operating characteristic curve, *CI* confidence interval, *LR* logistic regression, *SVM* support vector machines, *KNN* K- nearest neighbor, *XGBoost* eXtreme gradientbosting, *LightGBM* light gradient boosting machine, *MLP* multilayer perceptron

**Table 3:** Performance of SVM diagnostic models constructed based on different DLF

	AUC (95% CI)		Accuracy		Sensitivity		Specificity	
DL model	Training cohort	Validation cohort	Training cohort	Validation cohort	Training cohort	Validation cohort	Training cohort	Validation cohort
DenseNet121	0.996 (0.990–1.000)	0.951 (0.887–1.000)	0.978	0.912	1.000	0.875	0.958	0.944
DenseNet169	0.957 (0.922–0.992)	0.919 (0.798–1.000)	0.911	0.909	0.891	0.867	0.930	0.944
InceptionV3	0.990 (0.980–1.000)	0.956 (0.895–1.000)	0.948	0.909	0.968	0.875	0.931	0.941
ResNet18	0.996 (0.990–1.000)	0.920 (0.829–1.000)	0.978	0.882	0.984	0.750	0.972	1.000
ResNet50	0.990 (0.980–1.000)	0.886 (0.774–0.998)	0.948	0.848	0.968	0.812	0.931	0.882
ResNet101	0.978 (0.950–1.000)	0.890 (0.762–1.000)	0.941	0.879	0.889	0.750	0.986	1.000

### Performance of diagnostic models

**Fig. 4:**
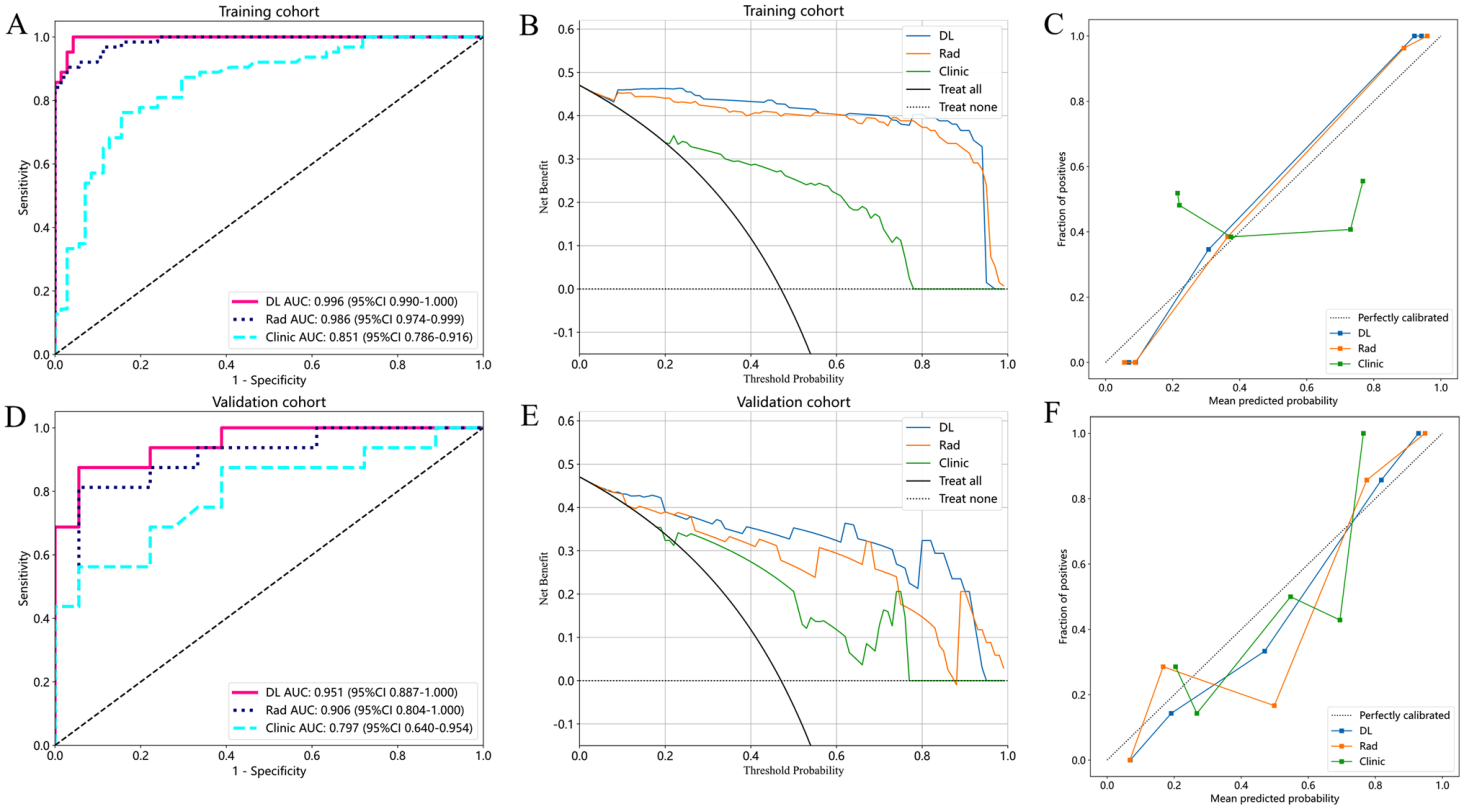
The performance evaluation of Clinic, RF and DL signatures. The ROC, DCA and calibration curves of the training (**A–C**) and validation cohort (**D–F**)

We developed three pre-procedural diagnostic models for predicting PCI using clinical data, RF, and DLF. It is worth noting that the AUC of both Rad and DL models was significantly higher than Clinic model. In the training cohort, the AUC of DL model was 0.996, which was higher than Clinic model (AUC = 0.851, *P* < 0.001) and Rad model (AUC = 0.986, *P* = 0.1468). In the validation cohort, the AUC of DL model was 0.951, which was higher than Clinic model (AUC = 0.797, *P* = 0.074) and Rad model (AUC = 0.906, *P* = 0.4654), as shown in Fig. 4A and D.

The results of the DCA demonstrated that patients could derive greater net benefits from DL model in comparison to both Clinic and Rad models (Fig. 4B and E). Additionally, the calibration curves indicated a superior level of agreement between predicted and actual groupings within DL models (Fig. 4C, F).

We also constructed a Clinic-Rad-DL model, which had an AUC of 0.962 (95% CI 0.901–1.000) in the validation cohort. However, the Clinic-Rad-DL model did not result in a substantial improvement in efficacy compared to DL model alone (*P* = 0.695).

**Fig. 5:**
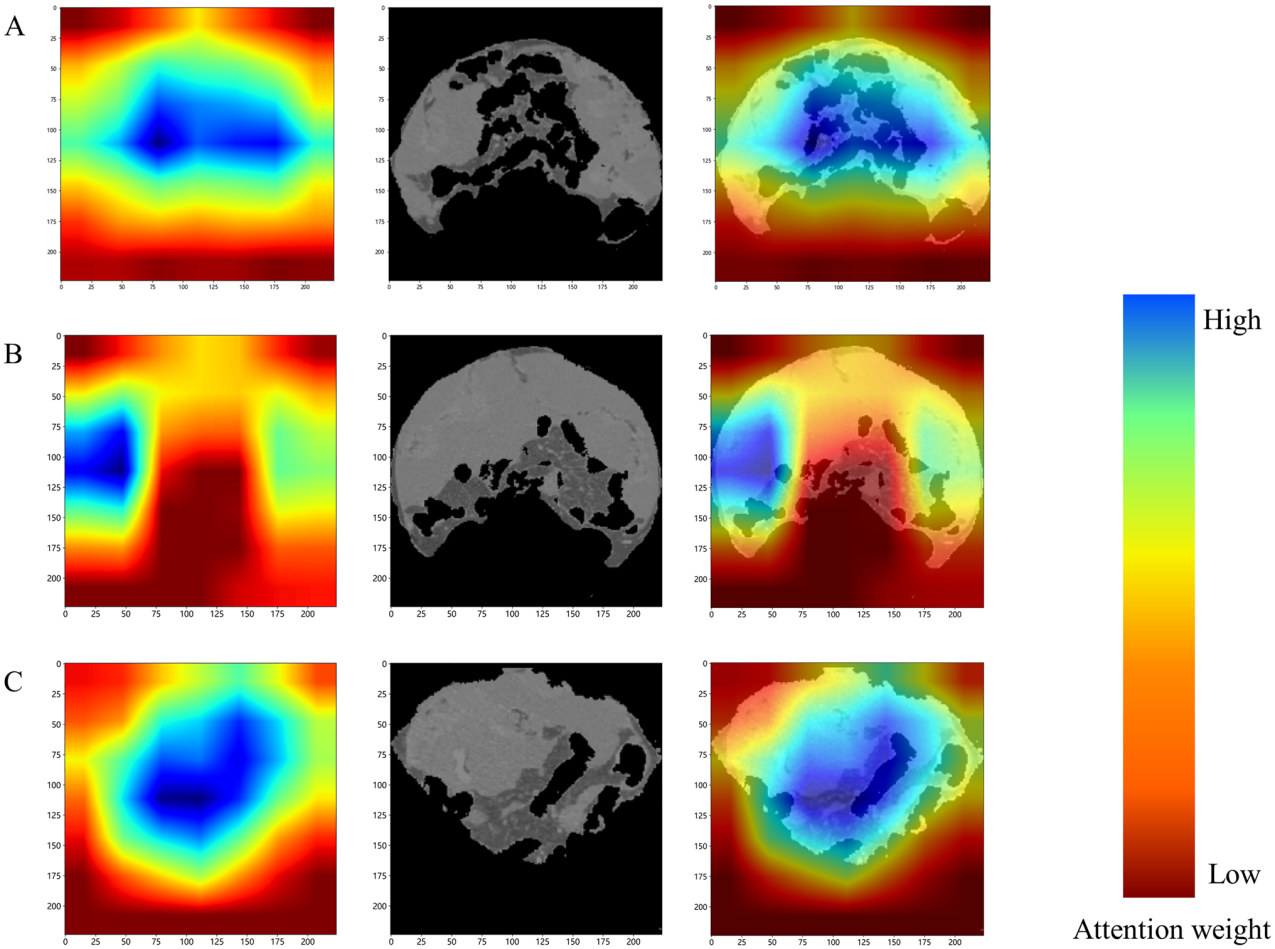
Grad-CAM visualization for DenseNet121 feature extraction. **A–C** shows the upper, middle and lower abdominal CTs of a patient with PM. The degree of contribution to the predicted results gradually increases from red to blue. (Color figure online)

While the features extracted by DL may be challenging to interpret in practical terms, the study implemented Grad-CAM to provide interpretability to the DL model. This involved characterizing the distribution of contributions to the output prediction results (Fig. 5).

Furthermore, we assessed the generalization ability of the DL model in practical applications by dividing patients into different subgroups. The DL model showed strong generalization ability in the stratified analysis according to age, gender, primary tumor, presence or absence of ascites, and different CT layer thicknesses (Table 4).

**Table 4:** Enhanced performance evaluation of the DL model across various subgroups

	Training cohort AUC (95% CI)	*P* value	Validation cohort AUC (95% CI)	*P* value
Age, years				
≤ 55	0.998 (0.941–1.000)	0.721	0.922 (0.676–0.996)	0.540
> 55	0.997 (0.941–1.000)		0.975 (0.773–1.000)	
Gender				
Female	0.995 (0.937–1.000)	0.853	0.962 (0.789–0.999)	0.937
Male	0.997 (0.940–1.000)		0.967 (0.664–1.000)	
Primary tumor				
Stomach	0.995 (0.936–1.000)	0.926	0.909 (0.660–0.994)	0.457
Others	0.995 (0.938–1.000)		0.974 (0.771–1.000)	
Ascites				
No (≤ 300 mL)	0.993 (0.954–1.000)	0.149	0.964 (0.808–0.999)	0.244
Yes (> 300 mL)	1.000 (0.872–1.000)		1.000 (0.631–1.000)	
Image thickness, mm				
1–1.5	1.000 (0.955–1.000)	0.227	0.983 (0.823–1.000)	0.347
5	0.990 (0.914–1.000)		0.875 (0.546–0.992)	

## Discussion

The purpose of this study was to develop and validate a DL model using preoperative enhanced abdominal CT to determine the degree of preoperative peritoneal involvement in PM patients. The results demonstrated that the model had satisfactory performance in stratifying patients and could potentially aid in treatment decision-making for PM patients.

Clinically, PM patients are usually at the end stage of the disease. Among the limited treatment options, CRS + HIPEC can maximize the overall survival time of patients, but this is only for patients with a small PM load [32, 33]. However, accurate preoperative staging of patients can avoid unnecessary surgical damage. Imaging examination, the most commonly used noninvasive diagnostic modality, has a limited ability to identify PM [34, 35]. CT combined with DL analysis in patients with PM has been widely used in predicting occult metastasis and recurrence of PM, demonstrating a strong predictive capability [27, 36, 37]. In a study of 163 patients, Zhang and colleagues [38] attempted to use radiomics rather than DL for preoperative PCI scoring, and used an external validation cohort to assess the applicability of the model. In their study, limited by intact peritoneal segmentation, only 6 regions were selected as representatives. In our study, we are the first to segment the complete peritoneum and use DL analysis of CT images for staging patients with preoperative PM. The results demonstrate the superior discriminatory performance of the Rad and DL models over clinical data alone. The DL model demonstrated superior AUC performance compared to the Rad model in both training and validation cohorts, while the DCA curve indicated that both models could enhance patient outcomes following CRS + HIPEC treatment. Additionally, Grad-CAM was used to address the black box problem of DL and provide clinicians with explanations. The focus on mesenteric heterogeneity in DL is consistent with what clinicians see intraoperatively, as the mesentery is a common site for PM.

In previous studies on using Rad or DL for PM, one of the main challenges was the segmentation of the ROI. Manual segmentation of ROI is subjective and can vary among different imaging physicians. Furthermore, the complexity of the peritoneum makes it difficult to manually sketch the entire peritoneum, and most studies [3, 27, 36, 39] only assess the status of the peritoneum in primary foci ROI or a portion of the peritoneum, which may not represent the entire peritoneum. Additionally, there is significant variation in the primary foci in patients with PM from numerous sources, and using only the primary foci ROI does not generalize the entire model to all patients with PM of tumor origin. To address these issues, we segmented the entire peritoneal region of patients semi-automatically using ITK-SNAP to segment the 3D peritoneal VOI for the first time. This approach allows us to accurately and comprehensively assess the peritoneal status of PM patients efficiently. Moreover, we divided the whole abdomen into three parts, and found that the majority of PM patients of gastric origin in our cohort and the majority of the finally screened RF and DLF also originated from the upper and middle abdomen. Clinically, gastric cancer PM is rarely found in the lower abdomen. This finding suggests that we can select different abdominal partitions for the classification of primary tumor origin to further optimize the efficacy of the model. Overall, the semi-automatic segmentation of the peritoneal VOI provides a more objective and comprehensive assessment of PM patients, and dividing the abdomen into different partitions may improve the model’s efficacy in identifying the primary tumor origin.

In our study cohort, a significant proportion of patients (47.0%) with S-PCI scores of 18 or higher underwent surgical exploration but did not receive any further surgical treatment, indicating that our DL model can potentially optimize available surgical options. Furthermore, our model holds promise for application in patients with higher PM load, where multi-stage HIPEC may be a viable option for achieving surgical indications similar to preoperative neoadjuvant chemotherapy [3]. As such, we anticipate that our model can enable selection of patients for surgical treatment and evaluation of HIPEC-treated SIP patients in the future.

However, there are limitations to our study that must be considered. First, fewer patients were evaluated for the S-PCI procedure, and larger samples may be needed to improve the reliability. Second, as our study was retrospective and based on a single-center cohort, the generalizability of the model must be validated with prospective and multicenter data. Finally, while DL analysis in medical imaging is primarily based on 2D imaging, the potential for future models utilizing 3D imaging analysis has the capacity to further optimize model performance.

In summary, our study presents a promising CT-based DL model for preoperative staging assessment of PM patients, providing a non-invasive means to optimize clinical treatment decisions.

### Electronic supplementary material

Below is the link to the electronic supplementary material.

Supplementary Material 1
