# Supplementary material for: CT-based deep learning model: a novel approach to the preoperative staging in patients with peritoneal metastasis
Source: Clin Exp Metastasis. 2023 Oct 5;40(6):493–504. doi: 10.1007/s10585-023-10235-5 (PMC10618318; doi:10.1007/s10585-023-10235-5)
Supplement: Supplementary file 1 — Supplementary Material 1 [file 10585_2023_10235_MOESM1_ESM.docx]

**Supplementary material**

**Supplementary 1：Sketching of peritoneal 3D volume of interest**

The patient's abdominal venous phase CT scan was segmented using ITK-SNAP software (version 4.0.0), with separate outlining of the upper, middle and lower abdomen. The upper, middle, and lower abdomen were distinguished based on anatomical landmarks such as the upper part of the liver, the lower edge of the rib arch, the upper edge of the sacroiliac joint, and the pubic symphysis. The window width was set at 700 Hu and the window position at 30 Hu. Semi-automatic outlining was performed using the Snake Inspector function, with a Lower threshold of -150 and Upper threshold of -50 to 0 in Segment 3D. Subsequently, the add bubble at cursor tool was used with 800-1200 iterations to refine the VOI. Two radiologists (Y.N.H. and J.P.W.) manually corrected any areas outside the abdominal cavity beyond the refinement stage. Finally, the peritoneal VOI was obtained after excluding organs, vessels, and cavities and covering all peritoneal regions.

**Supplementary 2：radiomics signature development**

To generate a radiomics signature, a total of 4683 imaging histology features were extracted from the upper, middle, and lower peritoneal VOI. These features were categorized into 7 groups: Shape Features, First Order Features, Gray-Level Co-occurrence Matrix (GLCM) Features, Gray-Level Dependence Matrix (GLDM) Features, Gray-Level Run-Length Matrix (GLRLM) Features, Gray-Level Size Zone Matrix (GLSZM) Features, and Neighborhood Gray-Tone Difference Matrix Features (NGTDM), with 1561 features per site. Supplementary figure S1 displays the proportion of each feature.

Subsequently, 1231 features with ICC <0.75 were excluded, and the remaining 3542 features were analyzed using Spearman's correlation coefficient. Only one feature was selected as a representative feature from a group of features with a correlation coefficient >0.9, resulting in 612 features. Finally, Lasso regression was used to screen for 35 features that significantly correlated with S-PCI, which were used to generate a radiomics signature. This signature allowed for the calculation of a radiomics score (Rad-score), using the following formula:

**Rad-score**=0.4701492537313442

+0.021207×upper_exponential_glcm_DifferenceVariance

-0.011931×upper_exponential_glszm_GrayLevelNonUniformityNormalized

+0.064012×upper_exponential_glszm_SizeZoneNonUniformity

+0.022498×upper_exponential_ngtdm_Strength

-0.016528×upper_gradient_glszm_ZoneVariance

+0.018790×upper_log_sigma_3_0_mm_3D_firstorder_Kurtosis

+0.054007×upper_log_sigma_3_0_mm_3D_glcm_Idn

-0.020510×upper_logarithm_glcm_Contrast

+0.053053×upper_original_firstorder_InterquartileRange

+0.000964×upper_original_shape_Maximum2DDiameterRow

+0.015229×upper_square_firstorder_Kurtosis

+0.027712×upper_square_glcm_JointEntropy

+0.064418×upper_wavelet_HLL_firstorder_Mean

-0.020194×upper_wavelet_HLL_firstorder_Range

-0.019242×upper_wavelet_HLL_glszm_LargeAreaHighGrayLevelEmphasis

-0.037869×upper_wavelet_HLL_ngtdm_Strength

-0.037641×upper_wavelet_LLH_firstorder_Maximum

+0.027948×middle_exponential_firstorder_Skewness

+0.101154×middle_exponential_gldm_SmallDependenceLowGrayLevelEmphasis +0.011049×middle_log_sigma_3_0_mm_3D_firstorder_Kurtosis

+0.083927×middle_logarithm_glcm_Correlation

+0.021768×middle_original_shape_MajorAxisLength

+0.003616×middle_square_firstorder_Range

+0.022898×middle_wavelet_HLL_glcm_Correlation

+0.004541×middle_wavelet_LHH_glcm_Idn

-0.030981×lower_log_sigma_2_0_mm_3D_glcm_Correlation

-0.008339×lower_log_sigma_3_0_mm_3D_glcm_ClusterShade

-0.002112×lower_log_sigma_3_0_mm_3D_glcm_Correlation

+0.001683×lower_logarithm_glszm_ZoneVariance

-0.005892×lower_wavelet_HHL_firstorder_Maximum

-0.038100×lower_wavelet_HHL_ngtdm_Strength

+0.032503×lower_wavelet_HLL_glcm_Correlation

-0.088073×lower_wavelet_LHH_firstorder_Median

+0.011018×lower_wavelet_LHH_glszm_LargeAreaHighGrayLevelEmphasis

-0.069772×lower_wavelet_LLL_firstorder_Skewness

**Supplementary 3：The architecture of DenseNet121**

We utilized the DenseNet121 model, which is based on ImageNet pre-training. The model consisted of an initial input layer of convolutional layers, 4 Dense blocks, 3 transition layers, and a total of 121 weight layers for the final output fully connected layer. The 4 Dense blocks comprised 6, 12, 24, and 16 1×1 +3×3 combined layers of 1×1 +3×3, respectively. Compared with the ResNet network structure, where only the parameters of the previous layer are input, the DenseNet121 input includes the parameters of the output of all the previous layers. This avoids the selective loss of features of some layers. The initial input of 224×224 images is reduced to 112×112 after the first layer of 7×7 convolution. The convolution of the Dense block and transition layer further reduces the size, and finally, the 1000 features of the fully connected layer are output after global average pooling and softmax. We chose to extract 1568 features of the penultimate layer features.denseblock4.denselayer16.conv2 for the next step of filtering and analysis.


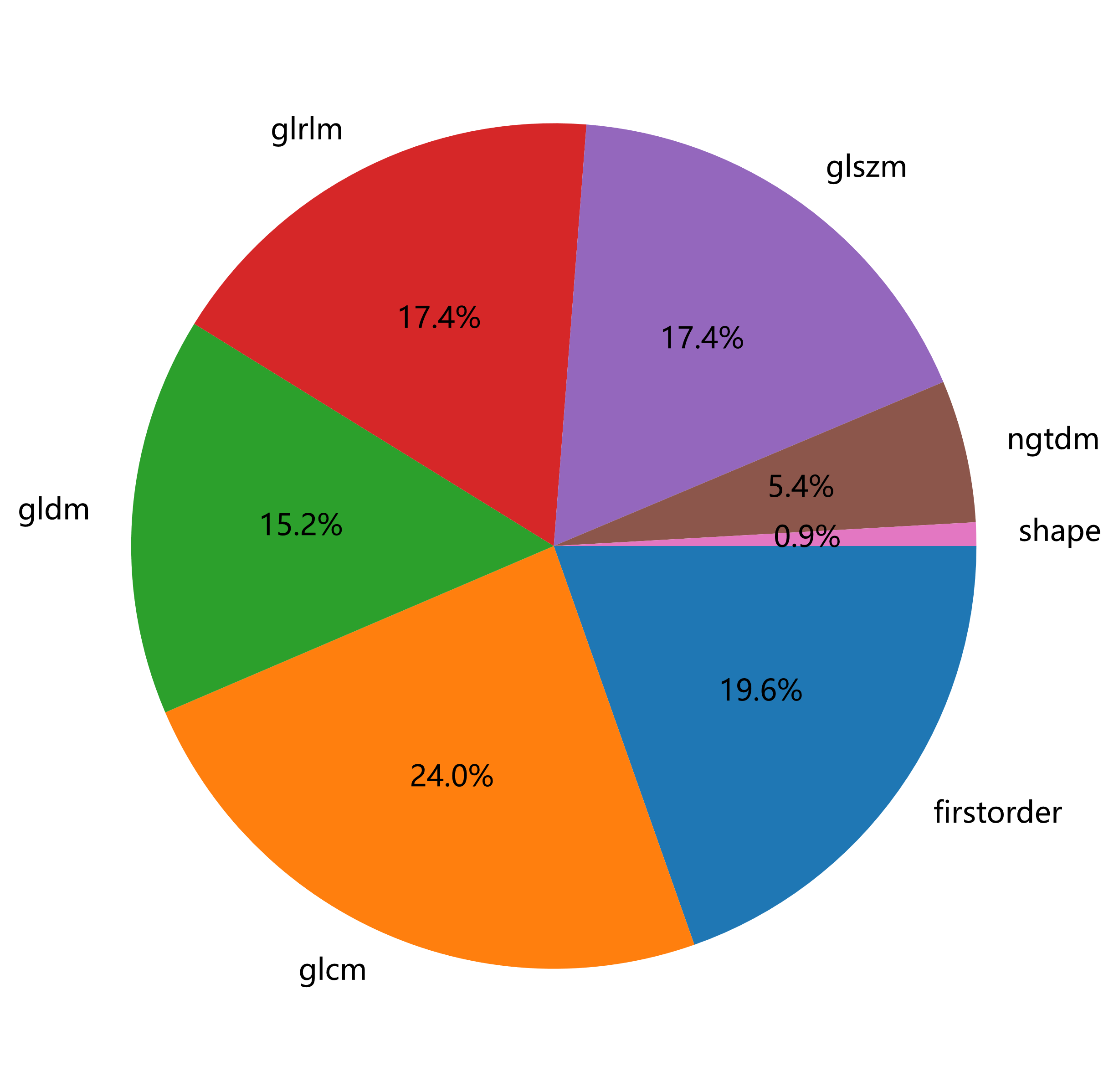


**Supplementary figure S1.** The category pie chart of all extracted radiomics features.
